# Cytogenetic analyses of eight species in the genus *Leptodactylus* Fitzinger, 1843 (Amphibia, Anura, Leptodactylidae), including a new diploid number and a karyotype with multiple translocations

**DOI:** 10.1186/1471-2156-13-109

**Published:** 2012-12-26

**Authors:** Thiago Gazoni, Simone L Gruber, Ana PZ Silva, Olivia GS Araújo, Hideki Narimatsu, Christine Strüssmann, Célio FB Haddad, Sanae Kasahara

**Affiliations:** 1 Departamento de Biologia, Instituto de Biociências, Universidade Estadual Paulista, UNESP, Rio Claro, São Paulo, Brazil; 2 Laboratório de Ecologia e Evolução, Instituto Butantan, São Paulo, São Paulo, Brazil; 3 Departamento de Zoologia, Instituto de Biociências, Universidade Estadual Paulista, UNESP, Rio Claro, São Paulo, Brazil; 4 Departamento de Ciências Básicas e Produção Animal, Faculdade de Agronomia, Medicina Veterinária e Zootecnia, UFMT, Cuiabá, Mato Grosso, Brazil

**Keywords:** FISH, Replication banding, BrdU, Fluorochrome staining, Ag-NOR, C-banding

## Abstract

**Background:**

The karyotypes of *Leptodactylus* species usually consist of 22 bi-armed chromosomes, but morphological variations in some chromosomes and even differences in the 2n have been reported. To better understand the mechanisms responsible for these differences, eight species were analysed using classical and molecular cytogenetic techniques, including replication banding with BrdU incorporation.

**Results:**

Distinct chromosome numbers were found: 2n = 22 in *Leptodactylus chaquensis*, *L*. *labyrinthicus*, *L*. *pentadactylus*, *L*. *petersii*, *L*. *podicipinus*, and *L*. *rhodomystax*; 2n = 20 in *Leptodactylus* sp*.* (aff. *podicipinus*); and 2n = 24 in *L*. *marmoratus*. Among the species with 2n = 22, only three had the same basic karyotype. *Leptodactylus pentadactylus* presented multiple translocations, *L*. *petersii* displayed chromosome morphological discrepancy, and *L*. *podicipinus* had four pairs of telocentric chromosomes. Replication banding was crucial for characterising this variability and for explaining the reduced 2n in *Leptodactylus* sp*.* (aff. *podicipinus*). *Leptodactylus marmoratus* had few chromosomes with a similar banding patterns to the 2n = 22 karyotypes. The majority of the species presented a single NOR-bearing pair, which was confirmed using Ag-impregnation and FISH with an rDNA probe. In general, the NOR-bearing chromosomes corresponded to chromosome 8, but NORs were found on chromosome 3 or 4 in some species. *Leptodactylus marmoratus* had NORs on chromosome pairs 6 and 8. The data from C-banding, fluorochrome staining, and FISH using the telomeric probe helped in characterising the repetitive sequences. Even though hybridisation did occur on the chromosome ends, telomere-like repetitive sequences outside of the telomere region were identified. Metaphase I cells from *L*. *pentadactylus* confirmed its complex karyotype constitution because 12 chromosomes appeared as ring-shaped chain in addition to five bivalents.

**Conclusions:**

Species of *Leptodactylus* exhibited both major and minor karyotypic differences which were identified by classical and molecular cytogenetic techniques. Replication banding, which is a unique procedure that has been used to obtain longitudinal multiple band patterns in amphibian chromosomes, allowed us to outline the general mechanisms responsible for these karyotype differences. The findings also suggested that *L*. *marmoratus*, which was formerly included in the genus *Adenomera*, may have undergone great chromosomal repatterning.

## Background

The genus *Leptodactylus* currently consists of 89 species that are distributed from the southern United States to Argentina [1]. The majority of these species occurs in the Neotropical region, and 67 have been recorded in Brazil [2].

Major changes have been introduced in the family Leptodactylidae because of the extensive taxonomic and systematic reviews that have occurred in the last years [3-5]. For example, the number of genera was reduced from 57 to only four, with representatives of *Adenomera* Steindachner, 1867, *Lithodytes* Fitzinger, 1843, and *Vanzolinius* Heyer, 1974 allocated in the genus *Leptodactylus*. Recently, other modifications have been suggested for Leptodactylidae by Pyron and Wiens [6], who considered the family as composed of 13 genera and again recognised *Adenomera* and *Lithodytes* as valid genera, even though synapomorphies and/or individual diagnosis have not been pointed out. Furthermore, the relationship between *Adenomera* and *Leptodactylus* remains a controversial issue [7,8].

Currently, approximately 40 species of *Leptodactylus*, *sensu* Frost et al. [3], have been karyotyped according to the revisions made by King [9], Kuramoto [10], Amaro-Ghilardi et al. [11], and Green and Sessions [12], complemented with subsequent information from Campos et al. [13] and Zaracho and Hernando [14]. The predominant diploid number is 2n = 22 and the karyotype constitution is considered conservative, including bi-armed metacentric, submetacentric, and subtelocentric chromosomes, which results in a fundamental number of chromosome arms of FN = 44. Nevertheless, a variable number of telocentric chromosomes has been reported in some karyotypes, which alters the FN. It is noteworthy that discrepant chromosome numbers, such as 2n = 18, 23, 24, and 26, are almost exclusively restricted to the former representatives of *Adenomera* and *Lithodytes*. The single known exception is *Leptodactylus silvanimbus* McCranie, Wilson and Porras, 1980, in which a diploid number of 2n = 24 was reported [11].

The first chromosome analyses on *Leptodactylus* were based exclusively on standard staining techniques. The first reports using differential staining did not appear until the 1990s, and it was not until many years later that molecular cytogenetic techniques were used [11,13-20]. However, studies using autoradiographic methods had been reported before [21,22]. Banding techniques have generated a larger number of markers that cytogenetically distinguish species or populations, but data on the chromosomal evolution of the genus *Leptodactylus* remain minimal.

This paper concerns the cytogenetic analyses of eight species of *Leptodactylus*, one of which has not yet been identified to species level. Taking into consideration that some of these species present distinct chromosome numbers or constitutions, both routine and molecular cytogenetic techniques were used. To better understand the mechanisms responsible for the karyotype variability observed within this genus, the present study emphasised the comparisons of the replication banding patterns using 5-bromodeoxyuridine incorporation.

## Methods

### Analysed species

All individuals were collected in the wild under governmental collection permits issued by the Instituto Chico Mendes de Conservação da Biodiversidade (ICMBio) to TG, OGSA, HN, CS, and CFBH. The euthanasia was performed by deep anesthesia under consent and approval of the ethics committee in animal use - CEUA (permission 005/2009), Instituto de Biociências, UNESP, Rio Claro, SP, Brazil.

Cytogenetic analyses were performed on 34 specimens: three *Leptodactylus chaquensis* Cei, 1950; three *Leptodactylus labyrinthicus* (Spix, 1824); seven *Leptodactylus marmoratus* (Steindachner, 1867); one *Leptodactylus pentadactylus* (Laurenti, 1768); two *Leptodactylus petersii* (Steindachner, 1864); nine *Leptodactylus podicipinus* (Cope, 1862); three *Leptodactylus rhodomystax* Boulenger, 1884; and six *Leptodactylus* sp*.* (aff. *podicipinus*), collected in the Brazilian states of Amapá (AP), Mato Grosso (MT), Mato Grosso do Sul (MS), Minas Gerais (MG), and São Paulo (SP) (Additional file 1). Almost all the voucher animals were deposited in the amphibian collection Célio F. B. Haddad (CFBH) housed in the Departamento de Zoologia, Instituto de Biociências, UNESP, Rio Claro, SP, Brazil, except one specimen of *L*. *labyrinthicus* collected in São Joaquim da Barra (SP), that was identified with the field number RJS 1420.

### Standard and molecular cytogenetic techniques

Direct chromosome preparations were obtained from bone marrow, liver, and testis and from intestinal epithelium [23,24]. For some animals, cell suspensions were obtained via lymphocyte cultures [25]. *In vitro* or *in vivo* treatments with 5-bromodeoxyuridine (BrdU) were used [16,25] to differentiate replication bands. Standard staining was performed with Giemsa, and differential staining was performed using the techniques of Ag-NOR [26], C-banding [27], Fluorochrome Plus Giemsa (FPG) [28], and DAPI and CMA_3_ fluorochrome staining [29]. The rDNA probe HM123 [30] was used in fluorescence *in situ* hybridisation (FISH) experiments [31] and a telomeric probe was used according to the manufacturer’s manual (Dako Cytomation Denmark A/S Kit). The bi-armed chromosomes were classified as metacentric, submetacentric, or subtelocentric and the chromosomes that were uni-armed were classified as telocentric [12,32].

## Results

### Karyotype constitution and meiosis

**Figure 1:**
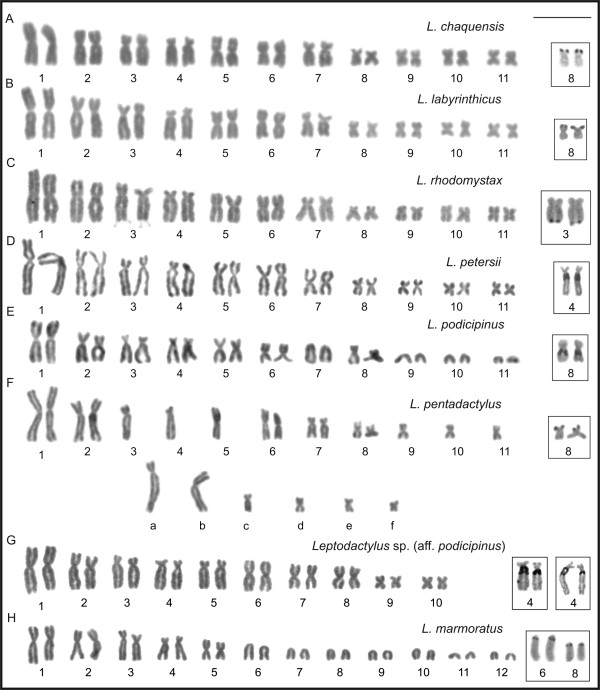
**Giemsa-stained karyotypes and chromosome pairs with Ag-NOR of *Leptodactylus*. A.***L*. *chaquensis*, male, 2n = 22; **B.***L*. *labyrinthicus*, male, 2n = 22; **C.***L*. *petersii*, male, 2n = 22; **D.***L*. *rhodomystax*, juvenile, 2n = 22; **E.***L*. *podicipinus*, female, 2n = 22; **F.***L*. *pentadactylus*, male, 2n = 22; **G.***Leptodactylus* sp*.* (aff. *podicipinus*), male, 2n = 20; **H.***L*. *marmoratus*, juvenile, 2n = 24. In **F**, the letters **a**, **b**, **c**, **d**, **e**, and **f** correspond to the rearranged chromosomes. Insets show chromosome pairs with Ag-NOR. Bar = 10 μm.

*Leptodactylus chaquensis*, *L*. *labyrinthicus*, *L*. *petersii*, and *L*. *rhodomystax* had 2n = 22, FN = 44, and karyotypes formed by seven large- and medium-sized pairs and four small pairs (Figure 1A-D). Among these, pairs 1, 5, 6, 9, 10, and 11 were metacentric; pairs 2, 3, 7, and 8 were submetacentric; and pair 4 was subtelocentric. Despite the submetacentric morphology, the chromosome 7 in *L*. *petersii* had greater relative length and distinct arm length ratio regarding those of the chromosome 7 of the remaining species. In the karyogram of *L*. *petersii*, chromosome 7 *was* the 5^th^ in size.

*Leptodactylus podicipinus* had 2n = 22, FN = 36, and a karyotype formed by seven large- and medium-sized pairs and four small pairs (Figure 1E). Among these, pairs 1, 5, and 6 were metacentric; pairs 2, 3, and 8 were submetacentric; pair 4 was subtelocentric; and pairs 7, 9, 10, and 11 were telocentric. *Leptodactylus pentadactylus* had 2n = 22, FN = 44, and an unusual karyotype (Figure 1F), in which only the chromosomes 1, 2, 6, 7, and 8 could be paired. Chromosomes 3, 4, 5, 9, 10, and 11, along with six others designated as **a**, **b**, **c**, **d**, **e**, and **f,** were unpaired elements. With exception of elements **a** to **f**, the *L*. *pentadactylus* chromosomes 1 to 11 were morphologically equivalent to chromosomes 1 to 11 observed in *L*. *chaquensis*, *L*. *labyrinthicus*, and *L*. *rhodomystax*. Chromosome **a** was subtelocentric; chromosomes **b** and **c** were submetacentric; and chromosomes **d**, **e**, and **f** were metacentric. *Leptodactylus* sp*.* (aff. *podicipinus*) had 2n = 20, FN = 40, and a karyotype formed by eight large- and medium-sized pairs and two small pairs (Figure 1G). Among these, pairs 1, 5, 6, 8, 9, and 10 were metacentric; pairs 2 and 3 were submetacentric; and pairs 4 and 7 were subtelocentric. In the karyogram of *Leptodactylus* sp*.* (aff. *podicipinus*) chromosome 7 was relatively large and was the 5^th^ in size. *Leptodactylus marmoratus* had 2n = 24, FN = 34, and a karyotype formed by six large- and medium-sized pairs and six small pairs (Figure 1H). Among these, pairs 1 and 5 were metacentric; pairs 2 and 3 were submetacentric; pair 4 was subtelocentric; and the remaining pairs 6 to 12 were telocentric.

Secondary constrictions were sporadically observed on chromosome 8 of *L*. *chaquensis*, *L*. *labyrinthicus*, *L*. *rhodomystax*, *L*. *pentadactylus*, and *L*. *podicipinus*, at the terminal short arm or, in the case of the latter species, at the proximal long arm. *Leptodactylus chaquensis* and *L*. *rhodomystax* also exhibited secondary constriction on the short arms of chromosome pairs 5 and 3, respectively. Chromosome 4 of *L*. *petersii* and *Leptodactylus* sp*.* (aff. *podicipinus*) showed secondary constriction at the proximal region on the long arm. *Leptodactylus marmoratus* occasionally showed this marker only at the proximal region of the long arm of the telocentric chromosome 6.

**Figure 2:**
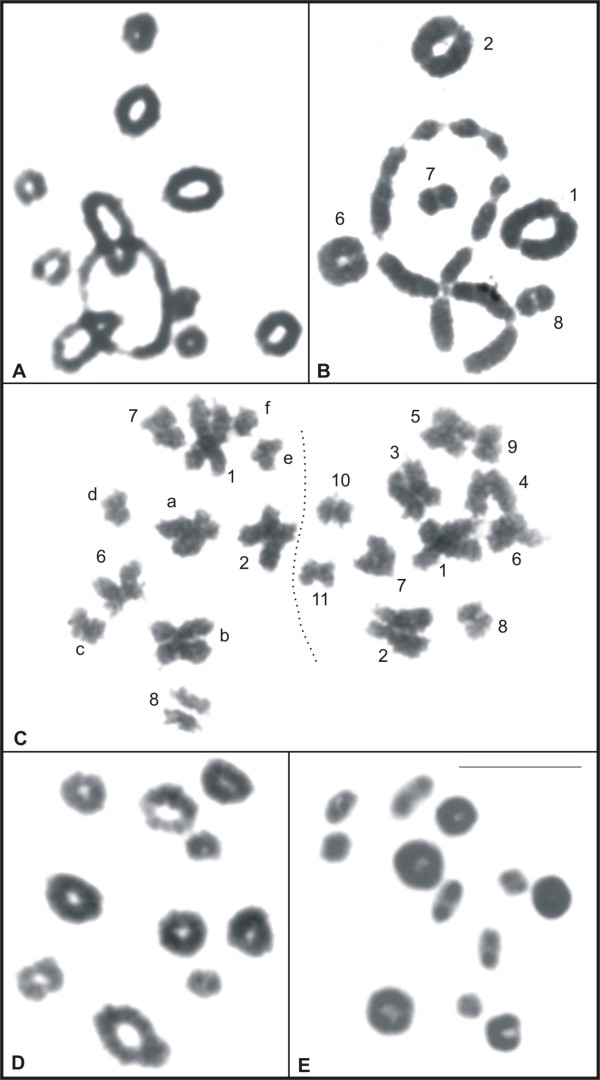
**Giemsa-stained meiotic cells of *Leptodactylus*. A.** metaphase I of *L*. *podicipinus*, with 11 bivalents; **B.** metaphase I of *L*. *pentadactylus*, with five bivalents and a ring-chain formed by 12 chromosomes; **C.** metaphases II of *L*. *pentadactylus,* showing 11 chromosomes, tentatively identified in each cell; **D.** metaphase I of *Leptodactylus* sp*.* (aff. *podicipinus*), with 10 bivalents; **E.** metaphase I of *L*. *marmoratus*, with 12 bivalents. In **C**, the letters **a**, **b**, **c**, **d**, **e**, and **f** correspond to the rearranged chromosomes. Bar = 10 μm.

Male meiotic cells from all species except *L*. *rhodomystax*, in which there was no adult male available, were analysed. The cells from species with karyotypes of 2n = 22, with exception of *L*. *pentadactylus*, had 11 bivalents during metaphase I, as shown for *L*. *podicipinus* in Figure 2A, and 11 chromosomes during metaphase II. Cells from *L*. *pentadactylus* in metaphase I had five bivalents, presumably corresponding to pairs 1, 2, 6, 7, and 8, and a ring-shaped chain formed by 12 chromosomes, presumably corresponding to the elements 3, 4, 5, 9, 10, 11, **a**, **b**, **c**, **d**, **e**, and **f** (Figure 2B). In metaphase II, 11 chromosomes were observed, and as seen in Figure 2C, the constitution of each cell could be distinguished. In both cells there was one element from the pairs 1, 2, 6, 7, and 8, and one of the cells contained additionally chromosomes 3, 4, 5, 9, 10, and 11, while the other cell contained the chromosomes **a**, **b**, **c**, **d**, **e**, and **f**. Cells in metaphase I from *Leptodactylus* sp*.* (aff. *podicipinus*) had 10 bivalents (Figure 2D) and cells in metaphase II had 10 chromosomes. Cells in metaphase I from *L*. *marmoratus* had 12 bivalents (Figure 2E) and cells in metaphase II had 12 chromosomes.

### Conventional banding and molecular cytogenetics

**Figure 3:**
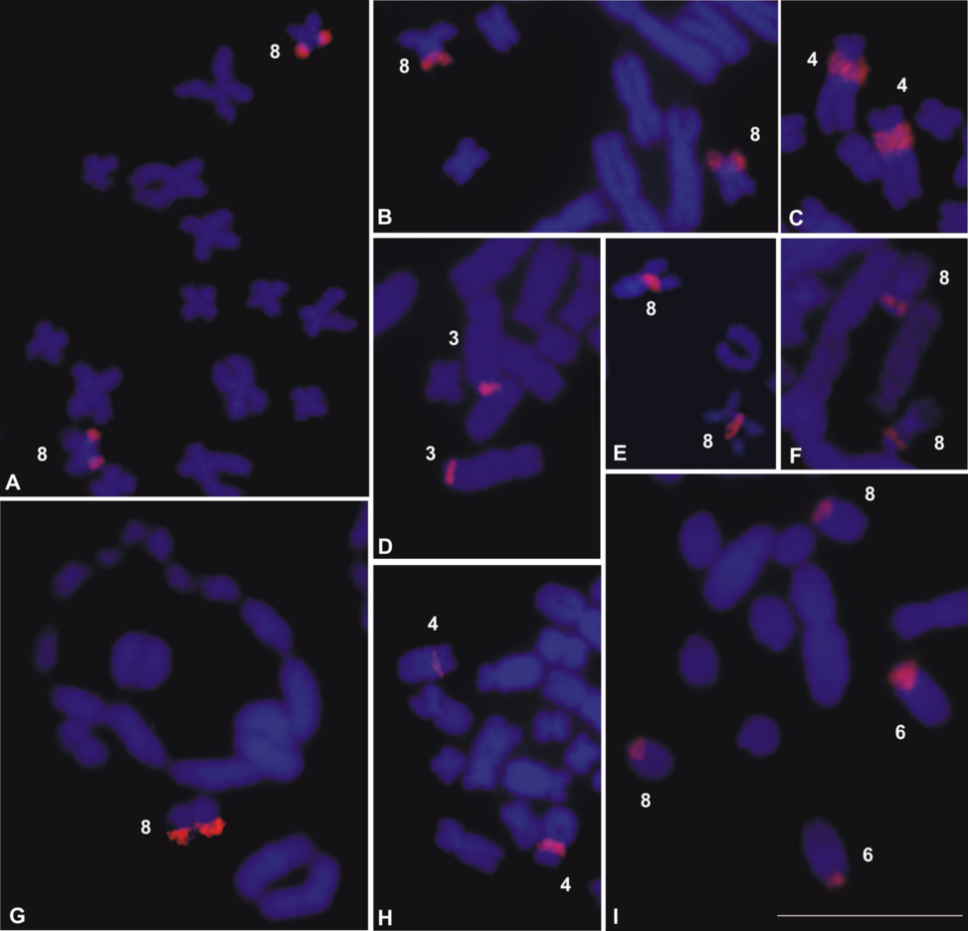
**FISH using an rDNA probe in *Leptodactylus*. A.** partial metaphase of *L*. *chaquensis*; **B.** partial metaphase of *L*. *labyrinthicus*; **C.** partial metaphase of *L*. *petersii*; **D.** partial metaphase of *L*. *rhodomystax*; **E.** partial metaphase of *L*. *podicipinus*; **F.** partial mitotic metaphase and **G.** metaphase I cell of *L*. *pentadactylus*; **H.** partial metaphase of *Leptodactylus* sp*.* (aff. *podicipinus*); **I.** partial metaphase of *L*. *marmoratus*. Bar = 10 μm.

The techniques of Ag-NOR (Figure 1) and FISH using an rDNA probe (Figure 3) revealed that there was a single NOR-pair located on chromosome pair 8 of *L*. *chaquensis*, *L*. *labyrinthicus*, *L*. *podicipinus*, and *L*. *pentadactylus*. In metaphase I cells from *L*. *pentadactylus* the rDNA probe hybridised to one of the bivalents, which identified it as the bivalent 8 (Figure 3G). In *L*. *rhodomystax*, the NOR was on chromosome 3, whereas in *L*. *petersii* and *Leptodactylus* sp*.* (aff. *podicipinus*) the NOR was on chromosome 4. Multiple NORs occurred in *L*. *marmoratus* that showed Ag-labelling and probe hybridisation at the proximal regions of the long arms of the chromosomes 6 and 8. Heteromorphic NORs were observed in *Leptodactylus* sp. (aff. *podicipinus*) and the larger Ag-NOR frequently appeared as a duplicated block (Figure 1G). FISH using an rDNA probe confirmed that the Ag-NOR heteromorphism was due to the size of the transcriptional segment and not to a differential genetic activity (Figures 1G, 3H). The sites of NOR were coincident with secondary constrictions in most cases.

**Figure 4:**
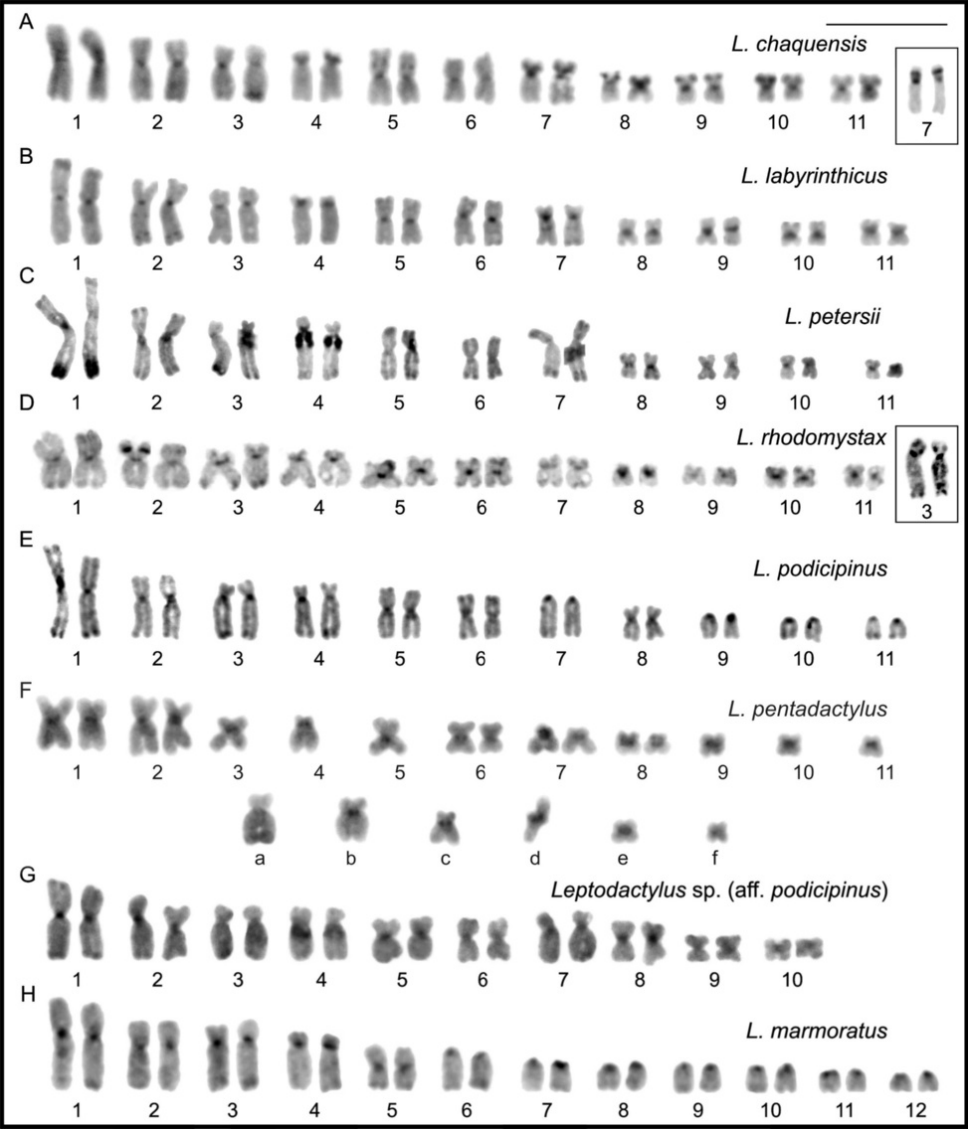
**C-banded karyotypes of *Leptodactylus*. A.***L*. *chaquensis*; **B.***L*. *labyrinthicus*; **C.***L*. *petersii*; **D.***L*. *rhodomystax*; **E.***L*. *podicipinus*; **F.***L*. *pentadactylus*; **G.***Leptodactylus* sp*.* (aff. *podicipinus*); **H.***L*. *marmoratus*. Inset shows in A chromosome pair 7 and in D chromosome pair 3 from another metaphases of *L*. *chaquensis* and *L*. *rhodomystax*, respectively. In **F**, the letters **a**, **b**, **c**, **d**, **e**, and **f** correspond to the rearranged chromosomes. Bar = 10 μm.

All of the *Leptodactylus* species had a predominantly centromeric distribution of C-banded heterochromatin (Figure 4). The NOR sites and, less frequently, the interstitial, terminal, or telomeric regions of some chromosomes also appeared to have C-band which was particularly evident in some species. For example, in *L*. *chaquensis*, C-bands were observed at the interstitial regions of the short arms of chromosomes 4 and 7, easily detected when the chromosomes were less condensed (Figure 4A). In *L*. *petersii*, C-bands were evident at the terminal long arm of chromosome 1 and in both terminal short and long arms of chromosome 7 (Figure 4C). In *L*. *rhodomystax*, C-bands were detected interstitially on the short arm of chromosome 2, in heteromorphic condition, and were occasionally detected at the interstitial short arm of chromosome 3 (Figure 4D). Furthermore, C-positive staining was also detected at the site coinciding with the negative heteropycnotic region on the short arms of the chromosome 5 in *L*. *chaquensis* and chromosome 8 in *L*. *rhodomystax*.

**Figure 5:**
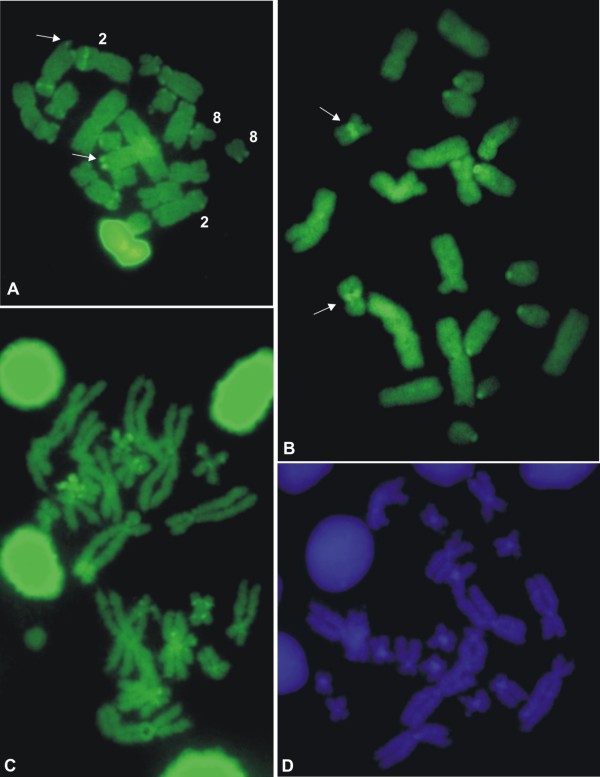
**Fluorochrome-stained metaphase cells of *Leptodactylus*. A.** CMA_3_ in *L*. *rhodomystax*; **B.** CMA_3_ in *L*. *podicipinus*; **C.** CMA_3_ and **D.** DAPI in *L*. *pentadactylus*. Arrows in A an B indicate NOR-bearing chromosomes. Bar = 10 μm.

The CMA_3_ staining exhibited brilliant fluorescence at the NOR sites for all of the species, as shown in Figure 5A-C for *L*. *rhodomystax*, *L*. *podicipinus*, and *L*. *pentadactylus*. These three species, along with *L*. *petersii* (data not shown), had additional CMA_3_ fluorescent labelling: in *L*. *rhodomystax*, at the interstitial short arms of chromosomes 2, 3, and other large- or medium-sized non-identified chromosomes and at the terminal region of a number of small-sized chromosomes, including the short arm of chromosome 8; in *L*. *podicipinus*, at the centromeric regions of all of the telocentric chromosome pairs and at the proximal region of chromosome 8; in *L*. *pentadactylus*, at the centromeric, interstitial, and terminal regions of some chromosomes, predominantly small-sized chromosomes; and in *L*. *petersii*, at the centromeric or terminal regions of some small-sized chromosomes, although the fluorescence was very faint. With DAPI staining, bright regions were not observed in most of the species. However, fluorescence was observed in the centromeric regions of some *L*. *pentadactylus* chromosomes (Figure 5D).

**Figure 6:**
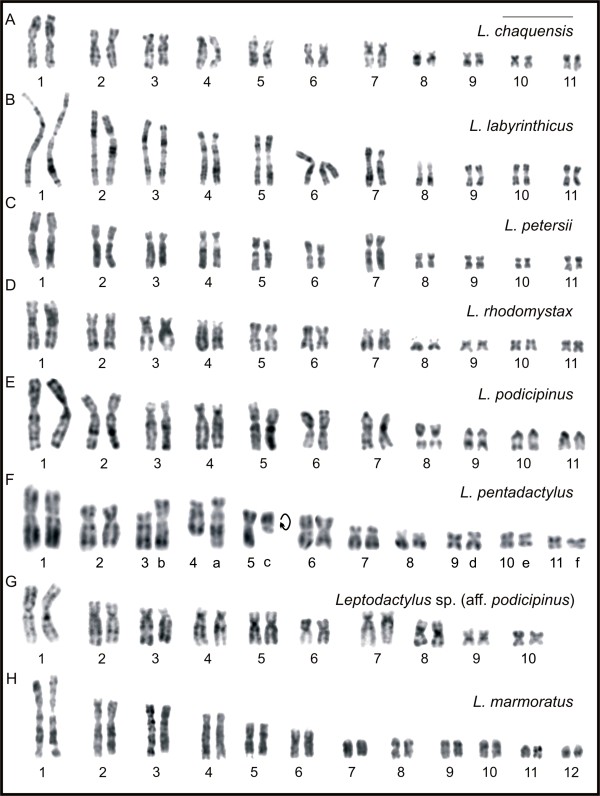
**Replication-banded karyotypes of *Leptodactylus*, after BrdU incorporation. A.***L*. *chaquensis*; **B.***L*. *labyrinthicus*; **C.***L*. *petersii*; **D.***L*. *rhodomystax*; **E.***L*. *podicipinus*; **F.***L*. *pentadactylus*; **G.***Leptodactylus* sp*.* (aff. *podicipinus*); **H.***L*. *marmoratus*. In **F**, the letters **a**, **b**, **c**, **d**, **e**, and **f** correspond to the rearranged chromosomes. Bar = 10 μm.

**Figure 7:**
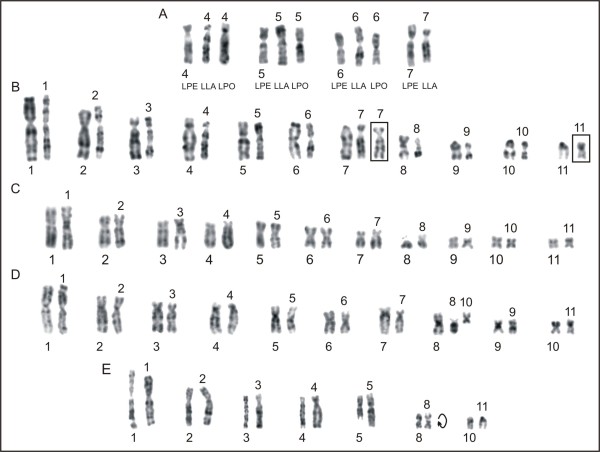
**Comparisons of replication-banded chromosomes of *Leptodactylus*. A.***L*. *petersii* (LPE), *L*. *labyrinthicus* (LLA), and *L*. *podicipinus* (LPO); **B.***L*. *podicipinus* (left) and *L*. *labyrinthicus* (right); **C.***L*. *pentadactylus* (left) and *L*. *rhodomystax* (right); **D.***Leptodactylus* sp*.* (aff. *podicipinus*) (left) and *L*. *chaquensis* (right); **E.***L*. *marmoratus* (left) and *L*. *podicipinus* (right)*.* In B, insets with chromosome 7 from another metaphase of *L*. *labyrinthicus* and chromosome 11 from *L*. *chaquensis*. Bar = 10 μm.

Using replication banding, homologous pairs were identified in each species (Figure 6). The replication banding patterns for chromosomes 1 to 11 were equivalent among the species *L*. *chaquensis*, *L*. *labyrinthicus*, and *L*. *rhodomystax*. Even though the banding differentiation in the small-sized chromosomes was poor, each of them had approximately the same patterns among the species. The comparison of the banded karyotype of *L*. *petersii* and of these three species also indicated no noticeable differences for the majority of the chromosomes. Figure 7A showed correspondence in the replication banding patterns of chromosomes 4, 5, and 6 in *L*. *petersii, L*. *labyrinthicus*, and *L*. *podicipinus*. There was also great banding correspondence between chromosomes 7 of *L*. *petersii* and *L*. *labyrinthicus*, but an additional late replicating band was visualised both in terminal short and long arms of the chromosome 7 in *L*. *petersii* (Figure 7A), corresponding to the heterochromatin region.

A comparison of banded chromosomes from *L*. *podicipinus* and *L*. *labyrinthicus* (Figure 7B) revealed that the replication banding patterns for chromosomes 1, 2, 3, 4, 5, 6, and 8 were equivalent between the two species. The uni-armed chromosomes 7, 9, 10, and 11 in *L*. *podicipinus* differed from the bi-armed chromosomes in *L*. *labyrinthicus* due to pericentric inversions. However, a better evidence of this rearrangement concerning the pair 11 was observed when the *L*. *podicipinus* chromosome 11 was compared with chromosome 11 of *L*. *chaquensis*.

**Figure 8:**
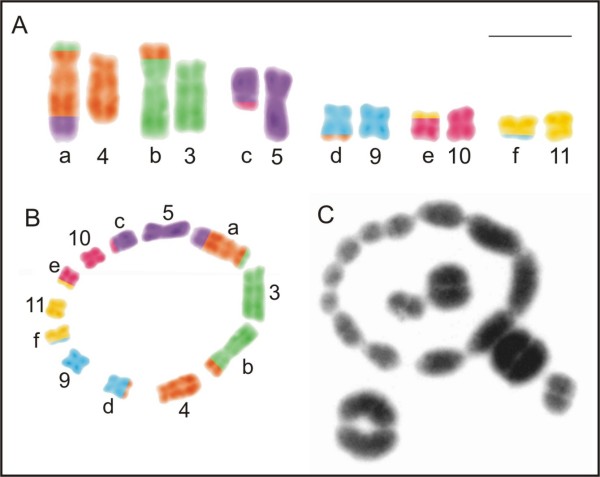
**Tentative identification of the multiple translocations in *Leptodactylus pentadactylus*. A.** schematic drawings of mitotic banded chromosomes and **B.** the chromosomes in meiotic chain; **C.** meiotic chain from metaphase I cell. The letters **a**, **b**, **c**, **d**, **e**, and **f** correspond to the rearranged chromosomes. Bar = 10 μm.

The banding pattern analysis for *L*. *pentadactylus* confirmed that chromosomes 1, 2, 6, 7, and 8 existed in pairs and identified the chromosomes 3, 4, 5, 9, 10, 11, **a**, **b**, **c**, **d**, **e**, and **f** as involved in rearrangements (Figure 6F). The chromosomes 1 to 11 of this species had the same replication banding patterns of the chromosomes 1 to 11 of *L*. *rhodomystax* (Figure 7C). In Figures 8A and 8B, the multiple translocations in *L*. *pentadactylus* and the position of these chromosomes in the meiotic ring-shaped chain (Figure 8C) were tentatively shown in schematic drawings.

The replication banding patterns on chromosomes 1, 2, 3, 4, 5, 6, 9, and 10 of *Leptodactylus* sp*.* (aff. *podicipinus*) (2n = 20) matched the patterns on chromosomes 1, 2, 3, 4, 5, 6, 9, and 11 of *L*. *chaquensis* (2n = 22), respectively (Figure 7D). The chromosomes 7 of these both species had equivalent banding pattern, but in *Leptodactylus* sp*.* (aff. *podicipinus*) the long arm of this chromosome was relatively longer than the long arm of the chromosome 7 in *L*. *chaquensis*. The long and short arms of chromosome 8 of *Leptodactylus* sp*.* (aff. *podicipinus*) matched the chromosomes 8 and 10 of *L*. *chaquensis*, respectively. As shown in Figure 7E, chromosomes 1 to 4 and chromosome 10 of *L*. *marmoratus* (2n = 24) had the same replication banding patterns as chromosomes 1 to 4 and chromosome 11 of *L*. *podicipinus* (2n = 22), respectively. Chromosomes 5 of both species had similar patterns, although partially, that is, chromosome 5 of *L*. *marmoratus* had correspondence with the short arm and proximal long arm of chromosome 5 of *L*. *podicipinus*. The telocentric chromosome 8 in *L*. *marmoratus* matched the submetacentric chromosome 8 in *L*. *podicipinus*, considering the chromosome of this latter species upside-down in Figure 7E.

**Figure 9:**
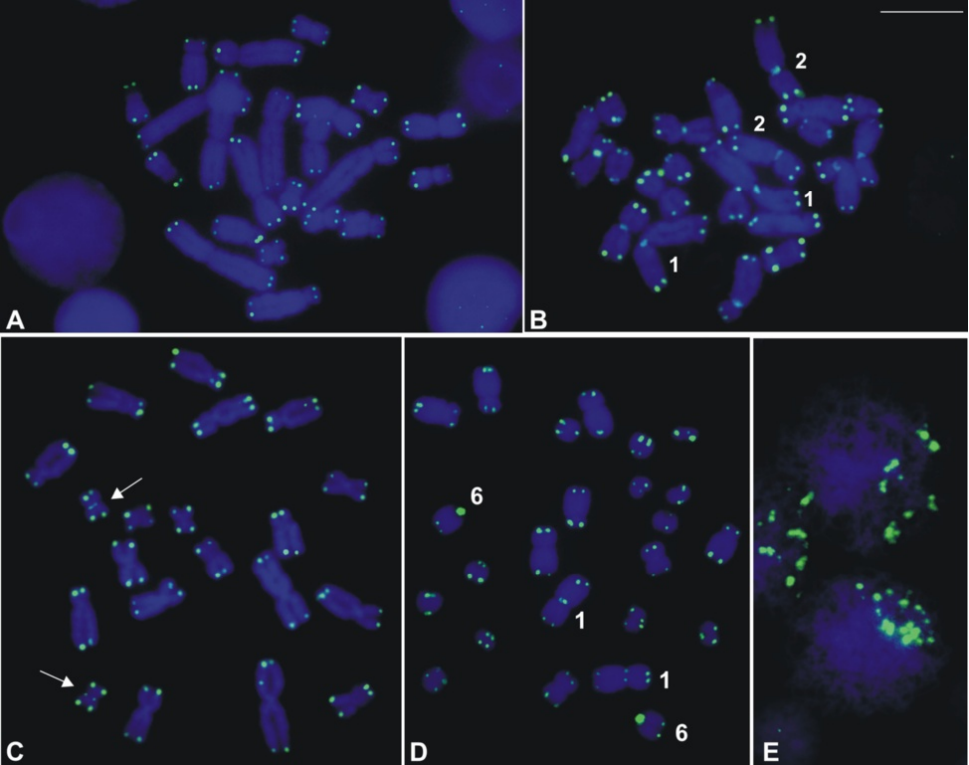
**FISH using a telomeric probe in *Leptodactylus*. A.** mitotic metaphase of *L*. *pentadactylus*; **B.** mitotic metaphase of *L*. *podicipinus*; **C.** mitotic metaphase of *Leptodactylus* sp*.* (aff. *podicipinus*); **D.** mitotic metaphase of *L*. *marmoratus*; **E.** initial meiotic nuclei of *L*. *pentadactylus* showing polarisation of the telomeric labelling. Note the centromeric hybridisation signals in chromosomes 1, 2, and other unidentified large- and medium-sized chromosomes in B; in chromosomes of one small-sized pair (arrows) in C; and in chromosomes 1 and in telocentric chromosomes 6 in D. Bar = 10 μm.

The telomeric probe hybridised with the chromosome ends in all species, as shown for *L*. *pentadactylus*, *L*. *podicipinus*, *Leptodactylus* sp. (aff. *podicipinus*), and *L*. *marmoratus* in Figures 9A-D respectively. In these three latter species, however, the chromosomes also exhibited hybridisation signals outside of the telomere region: for *L*. *podicipinus* in the centromeric regions on chromosomes 1 and 2 and on some of the other large- and medium-sized chromosomes; for *Leptodactylus* sp. (aff. *podicipinus*) in the centromeric region on one of small-sized chromosome pairs, even though the signal was very slight; and for *L*. *marmoratus* in the centromeric region on chromosome 1 and most probably in the centromeric region on telocentric pair 6. In meiotic preparations of *L*. *pentadactylus*, FISH with the telomeric probe could be obtained and in the initial meiotic nuclei fluorescent labelling appeared polarised as result of the bouquet configuration of the chromosomes (Figure 9E).

## Discussion

Among the analysed species of *Leptodactylus* six had 2n = 22 and two others had 2n = 20 or 2n = 24. Only *L*. *chaquensis*, *L*. *labyrinthicus*, and *L*. *rhodomystax* shared approximately the same basic karyotype of the genus with 22 bi-armed chromosomes. The similarity in the chromosome constitutions of these three species was also supported by the equivalence in the replication banding patterns of each chromosome pair. These data confirmed previous conclusions that, at least the largest chromosomes and the NOR-bearing chromosome 8 in several species had equivalent replication banding patterns [16-19]. Although *L*. *petersii* had the same 2n and FN of *L*. *chaquensis*, *L*. *labyrinthicus*, and *L*. *rhodomystax*, there was minor karyotype discrepancy regarding the relative size of pair 7. Nevertheless, the comparison of replication banding patterns confirmed the almost complete homeology between the chromosomes 7 of *L*. *petersii* and *L*. *labyrinthicus*. The difference was in the presence of an additional late replicating band, both in the short and in the long arms of chromosome 7 of *L*. *petersii*, which were shown to contain C-banded heterochromatin.

*Leptodactylus podicipinus* had an indistinguishable karyotype to those previously described for the species [16,20,33] and the most prominent feature was the presence of four pairs of telocentric chromosomes. Taking into consideration that the morphology of some chromosome pairs in *L*. *podicipinus* has been altered without changing the diploid number, it had been suggested that pericentric inversions might be responsible for such karyotype divergence [16]. Among the *Leptodactylus* species where 2n = 22 and some uni-armed chromosomes, *L*. *podicipinus* was the first case in which the replication banded telocentric chromosomes could be compared with the presumed homeologous bi-armed elements of *L*. *labyrinthicus*. The findings from the present analysis confirmed the hypothesis that pericentric inversion resulted in changes to the morphologies of chromosomes 7, 9, 10, and 11 in both species.

Even though *L*. *pentadactylus* had 2n = 22 with bi-armed chromosomes, the karyotype was one of the most intriguing, because only chromosomes 1, 2, 6, 7, and 8 could be accurately paired. With standard staining, chromosomes 3, 4, 5, **a**, **b**, **c**, and **f** did not have recognisable homologues and these four latter elements had no corresponding chromosomes identified among the species with basic karyotypes of 2n = 22 that matched them in either morphology or size. Chromosomes 9, 10, 11, **d**, and **e** could be tentatively paired based on morphological similarities, but one of them would remain without a homologue, supporting our suggestion that *L*. *pentadactylus* has a complex chromosome constitution. The meiotic analysis confirmed that multiple translocations are responsible for this unusual, but balanced karyotype. A ring-shaped chain formed by 12 chromosomes in addition to five bivalents in the metaphase I cells discarded the possibility of pairing between the repetitive sequences located in the terminal regions of the chromosomes. According to Schmid et al. [34], the non-chiasmatic ectopic pairing could be responsible for the formation of a meiotic chain observed in some analysed anuran species [35-37].

In natural populations of vertebrate, one example of species where meiotic chain was formed as result of multiple translocations is monotreme *Ornithorhynchus anatinus*. This species carries a multiple sex chromosome system of X_1_Y_1_X_2_Y_2_X_3_Y_3_X_4_Y_4_X_5_Y_5_:X_1_X_1_X_2_X_2_X_3_X_3_X_4_X_4_X_5_X_5_ type [38] and during meiosis of males alternate segregation occurs, which ensures balanced gametes with X or Y chromosomes. The chromosomes of the ring chain in *L*. *pentadactylus* male may undergo an alternate segregation, giving rise to two types of normal gametes, yet with rearranged chromosome constitution in one of them, as it was illustrated in Figure 2C. Our observation of two types of metaphase II cells, which likely originated from the same spermatocyte II, is according to an alternate segregation. Currently, however, adjacent segregations of the chromosomes have not been excluded and need to be investigated.

The replication banding pattern in the sampled *L*. *pentadactylus* collected from Paranaíta confirmed the uniqueness of the chromosome constitution, originated as a result of rare multiple rearrangements. An apparently normal karyotype with 22 bi-armed chromosomes was previously obtained for *L*. *pentadactylus* from both Peru and the state of São Paulo in southeastern Brazil [33]. Nevertheless, the sample from Brazil does not correspond to *L*. *pentadactylus* because its known distribution is limited to the Amazon forest in the northern part of South America [1]. In another study, a karyotype of 2n = 22 with heteromorphic reciprocal translocation was described for one juvenile specimen from Cláudia, a locality also in central Brazil, but authors [17] suggested that the rearrangement was produced during the fibroblast culture. Larger samples of *L*. *pentadactylus* from Paranaíta and vicinities, including specimens from Cláudia, should be karyotyped to test the hypothesis that heteromorphic multiple chromosome rearrangements are fixed or not in the populations, or whether other karyotype constitutions occur for the species.

The distinguishing feature in the karyotype of *Leptodactylus* sp. (aff. *podicipinus*) where 2n = 20 was the absence of two small-sized chromosome pairs and the presence of relatively larger chromosome pairs 7 and 8, when compared with the basic conserved *Leptodactylus* karyotype of 2n = 22. Correspondence between the replication banding patterns for the majority of the chromosomes of *Leptodactylus* sp. (aff. *podicipinus*) with the chromosomes of *L*. *chaquensis* where 2n = 22 was demonstrated. The comparative analysis confirmed the hypothesis that the reduction in the diploid number to 2n = 20 was the result of fusion between two small-sized elements, probably the chromosomes 8 and 10 in *L*. *chaquensis* giving rise to the chromosome 8 of *Leptodactylus* sp. (aff. *podicipinus*). The chromosomes 7 of both species had the same replication banding, but in *Leptodactylus* sp. (aff. *podicipinus*) the long arm of this chromosome is longer, may be because of the accumulation of repetitive sequences. Nevertheless, there was not evidence that these sequences were C-banded, as observed in the chromosome 7 of *L*. *petersii*.

To our knowledge, the karyotype with 2n = 20 of *Leptodactylus* sp. (aff. *podicipinus*) is new for the genus, not previously described. A detailed analysis, including characterisations of morphological traits, reproductive behaviours, vocalisations, geographical distribution, sequencing of molecular markers, and other characters of this taxon, should be conducted to investigate whether we are dealing or not with a new undescribed species. Interestingly, even though *Leptodactylus* sp. (aff. *podicipinus*) and *L. petersii* have distinct chromosome numbers, they have NORs located in the same site of the chromosome 4, this feature representing a synapomorphic condition for both species.

The karyotype of *L*. *marmoratus* was identical to those previously described [13,33] for specimens collected from the state of São Paulo. However, the first authors [13] did report population difference in morphology of the smallest chromosome pair, suggesting occurrence of pericentric inversion. Despite the similarities between the karyotypes of *L*. *marmoratus* (2n = 24) and *L*. *podicipinus* (2n = 22) regarding the first chromosome pairs and presence of telocentric chromosomes in both species, only a few chromosomes conserved the same replication banding patterns. These findings suggest that most of the chromosomes may have undergone great reorganization, which could not be detected in the banding comparisons. Nevertheless, the distinct chromosome numbers in both species most likely involved fusion between chromosome 5 and a small non-identified element in an ancestral karyotype equivalent to that of *L*. *marmoratus* or a chromosome fission of the chromosome 5 in an ancestral karyotype equivalent to that of *L*. *podicipinus*. Possible complex chromosome rearrangements or simple centromere repositioning which alters the chromosome morphology could not be identified because of the limited resolution of the techniques. An important question addresses the controversial systematics of *Adenomera* that, along with *Lithodytes*, were assigned within *Leptodactylus* according to the molecular phylogenetic trees of Frost et al. [3] and Grant et al. [4]. Recently, both were again considered to be valid genera of the family Leptodactylidae by Pyron and Wiens [6]. The molecular data by Silva et al. [unpublished data] support the first two reports recovering the monophyletic condition for *Leptodactylus* including *Adenomera* and *Lithodytes*. Even though the comparison of the replication-banded karyotypes of *L*. *marmoratus* and *L*. *podicipinus* could establish some chromosome homeology, it does not contribute to new insights into their chromosome evolution, which have been discussed in the literature [13,33,39].

In the sampled species, the combined use of silver impregnation and FISH using an rDNA probe confirmed that the majority of the secondary constrictions were active NORs. The negative heteropycnotic sites in chromosome 5 of *L*. *chaquensis* and in chromosome 8 of *L*. *rhodomystax*, which could indicate inactive nucleolar organiser regions, were excluded as true NORs. Both of the regions were C-positive and may represent species-specific repetitive sequence sites. A single pair of NORs occurs frequently among the *Leptodactylus* species, usually on the chromosome 8, although at distinct sites [11,16-18], as here observed in *L*. *chaquensis*, *L*. *labyrinthicus*, *L*. *pentadactylus*, and *L*. *podicipinus*. Less frequently, NORs are on large-sized chromosomes, such as the chromosome 3 in *L*. *rhodomystax* and the chromosome 4 in *L*. *petersii* and *Leptodactylus* sp. (aff. *podicipinus*). In *L*. *mystacinus*, NOR was found at the terminal short arm of chromosome pair 4, in addition to a NOR found on chromosome 8 [19]. In our samples, multiple NORs were confirmed in *L*. *marmoratus*, which had NORs located on telocentric chromosomes 6 and 8. This finding differed from previous data for this same species collected in distinct locations, in which a single Ag-NOR pair on chromosome 6 was observed, although one specimen showed an additional Ag-NOR on chromosome 8 [13]. Our data strongly suggest that the NOR on chromosome 8 may be an ancestral characteristic for the genus *Leptodactylus* and that even when the NOR is absent, as in *L*. *rhodomystax*, a vestige of this site remains, as evidenced by the C-banded heterochromatin at the short arm of chromosome 8, which showed brilliant CMA_3_ fluorescence.

Changes in the NOR site in *Leptodactylus* species were not the result of gross structural rearrangements because the chromosomes had the same replication banding patterns, regardless of whether they carried or not the rDNA sequence. Even the telocentric chromosome 8 of *L*. *marmoratus* had a replication pattern that was indistinguishable from the submetacentric chromosome 8 of *L*. *podicipinus*. The replication banding pattern of the chromosome 8 appears to be independent of the chromosome morphology and location of the NOR (*i*.*e*., at the short or long arm) which is characteristic of centromere repositioning. Nevertheless, minor structural rearrangements, such as reciprocal translocations or pericentric inversions, involving only the rDNA sequences, along with transpositions by mobile elements, cannot be disregarded.

The C-banding patterns were predominantly centromeric, although with some interstitial or terminal labelling, such as in *L*. *chaquensis*, *L*. *petersii*, and in *L*. *rhodomystax.* Interspecies differences in C-banding patterns, or even among distinct populations of the same species, may exist [16-18] although these findings should be considered with care because of variations in C-banding produced during technical procedures. In *L*. *chaquensis* males, a sub-centromeric C-band was not observed in either chromosome 1, discarding XY chromosome differentiation, as previously reported for the Argentinean specimens [15]. The cytogenetic information on repetitive sequences in the *Leptodactylus* species was improved by combining the C-banding technique with other procedures, such as stainings with AT- or GC-specific fluorochromes. These techniques not only revealed the molecular contents but also provided information on the occurrence of repetitive DNA sites, not detected by C-banding technique, as in the case of *L*. *pentadactylus*. In this species, although a centromeric C-banding pattern was noticed, CMA_3_ staining revealed repetitive sites out the centromeric region. Furthermore, the results using one or both fluorochromes evidenced that some patterns were species-specific, such as for *L*. *chaquensis*, *L*. *pentadactylus*, *L*. *petersii*, *L*. *podicipinus*, and *L*. *rhodomystax*. The FISH technique using a telomeric probe could be another useful tool for characterising the heterogeneity of some repetitive regions, such as in *L*. *marmoratus*, *L*. *podicipinus*, and *Leptodactylus* sp. (aff. *podicipinus*). In these species, the hybridisation signal was not only observed in telomere regions but was also in the centromeric regions of some chromosomes, which indicates that repetitive sequences similar to the telomeric sequence (TTAGGG)_n_ are present outside of the telomere-ends as it has been reported for other vertebrates, including frogs [40-43]. For all the remaining species of this study no interstitial telomeric signal was evident, even in the cases where structural rearrangements are presumed to have occurred during chromosome evolution, similarly to that observed in rodent species, whose karyotypes differed by fusion/fission events [44]. Nevertheless, the possibility that the centromeric labelling in a chromosome pair of small size, the 9 or the 10, in *Leptodactylus* sp. (aff. *podicipinus*) is a telomere remnant cannot be discarded because the corresponding chromosomes in some species of *Leptodactylus*, such as in *L*. *podicipinus*, differed by a pericentric inversion.

## Conclusions

Although the high karyotype similarity in most of the eight species of *Leptodactylus*, major and minor karyotype differences were evident using classical and molecular cytogenetic techniques. Discrepancies were observed in the morphology of some chromosomes, including the presence of telocentric chromosomes, the occurrence of multiple translocations, the distinct localisation of secondary constrictions, whether true NORs or not, and the molecular nature of some of the repetitive sequences. The replication banding after BrdU incorporation, which is one of the unique procedures to provide reproducible multiple bands throughout amphibian chromosomes, was fundamental for confirming the karyotype differences. This relatively little time-consuming technique allowed us to outline the mechanisms responsible for several karyotype differences, some of them never described before. Nevertheless, more species should be analysed using other approaches (*e*.*g*., cross-chromosome painting and linkage analysis), especially if they are combined with taxonomic data and phylogenetic trees based on distinct characters. Currently, the accumulation or loss of repetitive DNA sequences [45] cannot be ignored. Mapping this information on the chromosomes is essential for detailed karyotype comparisons and for enlightenment of chromosome evolution.

## Abbreviations

2n: Diploid number; Ag-NOR: Nucleolar organiser region marked by silver staining; BrdU: 5-bromodeoxyuridine; CMA_3_: Chromomycin A_3_; DAPI: 4’-6-diamidino-2-phenylindole; FISH: Fluorescence *in situ* hybridisation; FPG: Fluorochrome plus Giemsa; FN: Fundamental number of chromosome arms; NOR: Nucleolar organiser region; rDNA: Ribosomal DNA.

## Competing interests

The authors of this manuscript declare that they have no competing interests.

## Authors’ contributions

TG performed the cytogenetic analyses during his postgraduate course. SLG assisted in the FISH experiments and in the preparation of the final figures. APZS helped draft the manuscript and participated in the final revisions of the text. OGSA and HN collected animals and helped with identification. CS provided animals and revised the manuscript. CFBH helped with specimen identification and revisions to the manuscript. SK supervised the cytogenetic studies, the drafting of the manuscript, and the revision of the final text. All authors have read and approved the final text.

## Supplementary Material

Additional file 1 **List of karyotyped species of*Leptodactylus*, number of individuals, sex, voucher number, and collecting locations in Brazil.** (DOCX 12 kb) Click here for file
