# Supplementary material for: Cytogenetic analyses of eight species in the genus Leptodactylus Fitzinger, 1843 (Amphibia, Anura, Leptodactylidae), including a new diploid number and a karyotype with multiple translocations
Source: BMC Genet. 2012 Dec 26;13:109. doi: 10.1186/1471-2156-13-109 (PMC3562141; doi:10.1186/1471-2156-13-109)
Supplement: Additional file 1 — List of karyotyped species ofLeptodactylus, number of individuals, sex, voucher number, and collecting locations in Brazil. (DOCX 12 kb) [file 1471-2156-13-109-S1.docx]

# Additional file 1

## List of karyotyped species of Leptodactylus, number of individuals, sex, voucher number, and collecting locations in Brazil.

*Leptodactylus* *chaquensis* - two males, CFBH28569, CFBH28570, Três Lagoas, state of Mato Grosso do Sul (20º 45'S; 51º 40'W); one juvenile, CFBH28611, Nossa Senhora do Livramento, state of Mato Grosso (16º 14'S; 56º 22'W).

*Leptodactylus labyrinthicus -* two males, CFBH23257, CFBH28553, Rio Claro, state of São Paulo (22º 24'S; 47º 33'W); one female, RJS1420, São Joaquim da Barra, state of São Paulo (20º 34'S; 47º 51'W).

*Leptodactylus marmoratus* - three males, CFBH22416, CFBH22417, CFBH28592, one female, CFBH22235, and three juveniles, CFBH22409, CFBH22424 CFBH28642, Biritiba Mirim, state of São Paulo (23º 34'S ; 46º 02'W).

*Leptodactylus* *pentactylus* - one male, CFBH28618, Paranaíta, state of Mato Grosso (9º 39'S; 56º 28'W).

*Leptodactylus* *petersii* - one male, CFBH28645, Serra do Navio, state of Amapá (00º 53'N; 52º 00'W); one juvenile, CFBH28614, Paranaíta, state of Mato Grosso (9º 39'S; 56º 28'W).

*L*eptodactylus *podicipinus* - one male, CFBH28605, and two juveniles, CFBH28606, CFBH28607, Nossa Senhora do Livramento, state of Mato Grosso (16º 14'S; 56º 22'W); three females, CFBH28622, CFBH28623, CFBH28624, and two juveniles, CFBH28625, CFBH28626, Cáceres, state of Mato Grosso (16º 04'S; 57º 40'W); one male, CFBH28644, Gurinhatã, state of Minas Gerais (19º 12'S; 49º 46'W).

*Leptodactylus* *rhodomystax* - three juveniles, CFBH28565, CFBH28566, CBH28567, Paranaíta, state of Mato Grosso (9º 39'S; 56º 28'W).

*Leptodactylus* sp. (aff. *podicipinus*) - five males, CFBH28597, CFBH28598, CFBH28599, CFBH28600, CFBH28601, and one female, CFBH28602, Lucas do Rio Verde, state of Mato Grosso (13º 03'S; 55º 54'W).
